# The influence of persistent pathogens on circulating levels of inflammatory markers: a cross-sectional analysis from the Multi-Ethnic Study of Atherosclerosis

**DOI:** 10.1186/1471-2458-10-706

**Published:** 2010-11-17

**Authors:** Aydin Nazmi, Ana V Diez-Roux, Nancy S Jenny, Michael Y Tsai, Moyses Szklo, Allison E Aiello

**Affiliations:** 1 Department of Food Science and Nutrition, California Polytechnic State University, San Luis Obispo, CA, USA; 2 Department of Epidemiology, University of Michigan School of Public Health, 1415 Washington Heights, Room 3659, Ann Arbor, MI 48109, USA; 3 Department of Pathology, University of Vermont College of Medicine, Burlington, VT, USA; 4 Department of Laboratory Medicine and Pathology, University of Minnesota, Minneapolis, MN, USA; 5 Department of Epidemiology, Johns Hopkins Bloomberg School of Public Health, Baltimore, MD, USA

## Abstract

**Background:**

Systemic inflammation is linked to cardiovascular risk, but the influence of persistent pathogens, which are conventionally dichotomously categorized, on circulating levels of inflammatory markers is not clear. Antibody levels of pathogens have not been examined in relation to inflammation.

**Methods:**

Using data from a subsample of the Multi-Ethnic Study of Atherosclerosis, we examined circulating levels of interleukin-6 (IL-6), C-reactive protein (CRP) and fibrinogen in relation to five common persistent pathogens: cytomegalovirus, herpes simplex virus-1, Hepatitis A virus, *Helicobacter pylori *and *Chlamydia pneumoniae*. We tested the hypothesis that the number of seropositive pathogens (based on conventional cut-off points) would not be as sensitive a marker of inflammation as immune response measured by antibody levels to pathogens.

**Results:**

High antibody response to multiple pathogens showed graded and significant associations with IL-6 (p < 0.001), CRP (p = 0.04) and fibrinogen (p = 0.001), whereas seropositive pathogen burden did not. In multiple linear regression models, high antibody response to multiple pathogens maintained a positive association only with IL-6 (4.4% per pathogen exhibiting high antibody response, 95% CI 0.0-8.9).

**Conclusions:**

High antibody response to pathogens was a more consistent marker of inflammatory outcomes compared to seropositivity alone and high antibody response to multiple pathogens was a stronger marker compared to any single pathogen.

## Background

Persistent pathogens, those acquired early in life and maintained without causing obvious illness, are implicated in cardiovascular disease etiology. Numerous studies have suggested that persistent viruses such as cytomegalovirus (CMV), herpes simplex virus-1 (HSV), Hepatitis A virus (HAV) and bacterial pathogens such as *Helicobacter pylori *(*H. pylori*) and *Chlamydia pneumoniae *(*C. pneumoniae*) are associated with cardiovascular disease [1-4], although some studies do not support a significant relationship [5-7]. Effects of multiple infectious agents may be synergistic, and some authors suggest that pathogen burden (total number of pathogens) has a greater impact on cardiovascular risk than isolated pathogens [4,8,9]. It is hypothesized that the association between pathogens and cardiovascular disease is, in part, mediated through chronic activation of inflammatory pathways [10,11].

In parallel to work linking chronic infections to atherosclerosis, a number of studies have shown that various markers of systemic inflammation are linked to cardiovascular risk. Cytokines such as interleukin-6 (IL-6) induce the production and secretion of acute-phase proteins including C-reactive protein (CRP) and fibrinogen [12,13]. Chronic activation of these inflammatory pathways is hypothesized to promote atherogenesis and thrombosis [14]. Levels of IL-6 [15,16], CRP [17-19] and fibrinogen [20] show consistent associations with incident coronary events and subclinical disease independent of established cardiovascular risk factors. A number of risk factors for chronic inflammation (including smoking [21], physical activity [22], and obesity [23]) have been identified. However, the influence of persistent pathogens on circulating levels of inflammatory markers is unclear and existing studies have been inconsistent [24-26].

We used data from a large population-based sample to examine associations between infectious agents and inflammatory pathways implicated in cardiovascular disease in healthy adults. We examined circulating levels of IL-6, CRP and fibrinogen in relation to the presence of and antibody response to five pathogens: CMV, HSV, HAV, *H. pylori *and *C. pneumoniae*. We hypothesized that the conventional definition of pathogen burden (number of seropositive pathogens using conventional cut-off points) would not be as sensitive a marker of inflammation as an alternative definition also taking into account antibody levels of pathogens.

## Methods

### Study sample

The Multi-Ethnic Study of Atherosclerosis (MESA) is a population-based longitudinal study designed to investigate risk factors for atherogenesis. Study participants (n = 6814), aged 45 to 84 years, were recruited from six US communities and were free of clinical cardiovascular disease at the time of the baseline visit from July 2000 to September 2002. The current cross-sectional analysis uses data on *C. pneumonia *from the total cohort at baseline, and from a sub-sample of 1000 randomly selected cohort members who underwent serum testing for additional pathogens implicated in cardiovascular disease (CMV, HSV, HAV and *H. pylori*). Complete information including data on all infectious agents, inflammatory markers and covariables was available for 999 individuals. The Institutional Review Board at each participating site reviewed the study and written informed consent was collected from all participants. Detailed methods and aims of the MESA cohort are available [27].

### Data

During the baseline visit, a range of sociodemographic, behavioral and anthropometric variables were collected. Data included education categorized into four groups (less than high school, high school diploma or equivalent, some college or technical school and college diploma); body mass index (BMI, kg/m^2^) to define overweight or obesity status as ≥ 25 kg/m^2^; current alcohol intake; pack-years of cigarette smoking, current use of medications known to alter inflammatory levels (any one or more of the hormone replacement therapies, aspirin, oral anti-inflammatory agents, lipid-lowering drugs and non-steroidal anti-inflammatory drugs, grouped into a single dichotomous variable); and self-rated health measured on a scale of 1 (best) to 5 (worst); 1-3 were considered "good self-rated health".

### Lab tests

Serum IgG antibodies to CMV, HSV, and *H. pylori*, were measured by indirect enzyme immunoassay using commercially available kits (DiaMedex Corp. Miami, FL). The sensitivity and specificity of the tests ranged from 94-100%. Hepatitis A antibody was measured using the IMx HAVAB qualitative microparticle enzyme immunoassay (Abbott Laboratories, Abbott Park, IL). Immunoglobulin G antibodies to *C. pneumoniae *were detected using a microimmunoflourescent antibody assay with a range of 0-4 Microimmunofluorescence (MIF) units (Focus Technologies, Cypress, CA). Conventional cut-off points were used to define dichotomous seropositivity; equivocal values were classified as positive. Individuals were considered seropositive for CMV infection above 10.0 EU/mL, HSV infection above 16.0 EU/mL, HAV below a standardized calibrated rate, and *H. pylori *infection above 0.90 EU/mL [28]. Infection with *C. pneumoniae *was considered positive at 1 MIF unit or higher. We defined 'high antibody response' to pathogens as IgG values in the top quartile (lowest quartile for HAV). For *C. pneumoniae*, titer groups 3-4 were considered high antibody response.

Interleukin-6 (pg/mL) was measured by ultrasensitive ELISA (R&D Systems, Minneapolis, MN). CRP (mg/L) and fibrinogen (mg/dL) were assessed by nephelometry (BNII nephelometer, Dade Behring, Deerfield, IL). Analytic coefficients of variation were 6.3%, 2.6%, and 3.6% for IL-6, fibrinogen, and CRP, respectively.

### Statistical methods

Descriptive analyses were carried out using chi-squared tests (for trend where applicable). Kruskal-Wallis rank tests were performed for descriptive variables with skewed distributions. The dependent variables were circulating levels of IL-6 in pg/mL, CRP in mg/L and fibrinogen in mg/dL. Interleukin-6 and CRP presented skewed distributions and were log-normalized prior to analysis. Independent variables took two forms: 1) seropositive pathogen burden, defined as the count sum of seropositive pathogens using conventional cut-off points (0-5 pathogens with groups 0-1 collapsed for some analyses); and 2) high antibody response to multiple pathogens, defined as the count sum of pathogens with antibody response in the highest quartile (high antibody response to 0-5 pathogens, groups 3-5 collapsed for some analyses). We first examined associations of interleukin-6 (IL-6), C-reactive protein (CRP) and fibrinogen with dichotomous measures of high antibody response to each pathogen and second, with the number of pathogens for which there was a high response (0-5). We performed the same analysis using conventional cut-offs for seropositivity to each pathogen and the number of seropositive pathogens.

Tetrachoric (for dichotomous variables) correlation coefficients were calculated to investigate correlations between the different dependent variables and one-way ANOVA was used to test for differences in inflammatory levels according to levels of markers. Linear regression was used to estimate adjusted associations of seropositivity and high antibody levels with inflammatory markers. Four levels of covariate adjustment were used in regression models: 1) adjusted for age and sex; 2) adjusted for age, sex and race/ethnicity; 3) adjusted for age, sex, race/ethnicity and education; and 4) adjusted for all of the above plus body mass index, current alcohol intake, cigarette smoking (pack-years), diabetes, current use of medication and self-rated health.

## Results

The mean age of the sample was 59 years (range 44-84), with 46% white, 21% black, 23% Hispanic and 10% Chinese. Women comprised 57% of the sample. Eighteen percent had finished high school but had no college education and 29% and 37% of participants had some college education or completed college, respectively. Mean (SD) BMI was 28.6 kg/m^2 ^(5.6) and 72% were overweight or obese. Almost 90% rated their health as good.

Nearly 77% of the sample was seropositive for CMV, 85% for HSV, 58% for HAV, 46% for *H. pylori*, and 71% for *C. pneumoniae*. Less than 2% of the sample was negative for all five of the tested pathogens, whereas 51% were seropositive for either four or five. Tetrachoric correlation coefficients for seropositivity among pathogens ranged between 0.42-0.55 except for those involving *C. pneumoniae*, which were weaker and on the order of 0.12-0.24. All were statistically significant at the p < 0.05 level. Median (IQR) levels for IL-6, CRP, and fibrinogen were 1.12 pg/mL (0.72-1.74), 2.00 mg/L (0.82-4.59), and 340 mg/dL (296-391), respectively.

Table 1 shows the distribution of sociodemographic and health characteristics of the 999-participant sample according to number of seropositive pathogens. There was a positive trend of increasing age by number of pathogens. Non-whites and less educated individuals had higher seropositive pathogen burden (both p < 0.001). Alcohol consumption was associated with lower seropositive pathogen burden, as was self-rated good health, whereas smoking and diabetes were associated with a higher pathogen burden.

**Table 1:** Mean (SD) or percent sociodemographic and health characteristics according to seropositive pathogen burden in a sub-sample of the Multi-Ethnic Study of Atherosclerosis (2000-2002).

	Number of seropositive pathogens					P value*
	**0-1**	**2**	**3**	**4**	**5**	

Age, y	56.8 (8.4)	59.1 (9.7)	59.5 (10.0)	59.9 (10.1)	59.9 (9.6)	0.02
Female	54.6	54.6	58.5	59.4	56.1	0.8
Race/ethnicity						< 0.001
White	21.6	28.8	26.6	14.8	8.3	
Black	2.9	10.1	23.4	34.0	29.7	
Hispanic	1.7	3.9	12.9	35.3	46.1	
Chinese	1.0	1.0	16.2	35.4	46.5	
Education						< 0.001
< High school	0.0	1.2	15.1	34.9	48.8	
Finished high school	5.7	11.3	20.3	27.7	35.0	
Some college	8.4	16.8	25.5	26.6	22.7	
College graduate	20.7	25.3	22.6	19.6	12.0	
BMI, kg/m^2^	28.1 (5.4)	27.9 (5.3)	28.8 (6.1)	28.4 (5.5)	28.6 (5.7)	0.2
Smoking, pack years	12.2 (30.6)	10.0 (17.1)	18.4 (30.1)	9.8 (18.8)	8.0 (14.4)	0.03
Alcohol consumer	84.6	73.3	61.8	48.8	44.4	< 0.001
Diabetic	6.4	8.6	7.4	15.6	14.6	0.006
Self-rated good health	95.4	93.6	90.9	85.1	78.1	< 0.001
N	110	163	217	256	253	--

Pathogens examined:

*P-values by chi-squared test (sex, race/ethnicity, education, alcohol use, diabetes, self-rated health), chi-square test for trend (age and BMI), and Kruskal-Wallis test (pack-years of smoking)

BMI: body mass index, kg/m^2^

Alcohol consumer: alcoholic drink within the past week

Table 2 shows inflammatory marker levels by seropositivity and level of antibody response to each pathogen. Seropositivity for individual pathogens was associated with higher inflammatory levels for 11 of the 15 tested associations (all except HAV with CRP and fibrinogen; *H. pylori *with CRP; and *C. pneumoniae *with fibrinogen), although only one (HSV with fibrinogen) was significant (p = 0.006). HAV infection was significantly associated with fibrinogen as well, but inversely. High antibody response to multiple pathogens, on the other hand, was consistently associated with higher inflammatory levels across all markers, although only four (CMV with all three markers and *C. pneumonia *with IL-6) were significant.

**Table 2:** Inflammatory marker levels by seropositivity and high antibody response to each pathogen in a sub-sample of the Multi-Ethnic Study of Atherosclerosis (2000-2002).

Pathogen	Seropositivity* or antibody response^†^	N	IL-6, pg/mL	CRP, mg/L	Fibrinogen, mg/dL
Cytomegalovirus	Negative	227	1.07 (1.04)	1.90 (1.08)	345 (75)
	Positive	758	1.18 (1.02)	2.01 (1.05)	349 (73)
	P-value	-	0.06	0.5	0.5
	
	Q1-Q3	510	1.13 (1.03)	1.83 (1.06)	344 (75)
	Q4	249	1.31 (1.04)	2.44 (1.08)	360 (69)
	P-value	-	0.004	0.002	0.002

Herpes simplex virus-1	Negative	150	1.05 (1.05)	1.67 (1.10)	333 (65)
	Positive	835	1.18 (1.02)	2.04 (1.04)	351 (75)
	P-value	-	0.05	0.06	0.006
	
	Q1-Q3	600	1.15 (1.03)	2.02 (1.05)	350 (78)
	Q4	248	1.24 (1.04)	2.09 (1.08)	353 (68)
	P-value	-	0.2	0.7	0.4

Hepatitis A virus	Negative	425	1.14 (1.03)	2.02 (1.06)	353 (76)
	Positive	574	1.17 (1.03)	1.95 (1.05)	342 (70)
	P-value	-	0.6	0.7	0.02
	
	Q2-Q4	319	1.13 (1.04)	1.85 (1.07)	348 (73)
	Q1	255	1.21 (1.04)	2.10 (1.08)	357 (79)
	P-value	-	0.2	0.2	0.2

*H. pylori*	Negative	531	1.13 (1.03)	1.99 (1.06)	347 (76)
	Positive	454	1.18 (1.03)	1.97 (1.06)	350 (72)
	P-value	-	0.3	0.9	0.5
	Q1-Q3	215	1.17 (1.04)	1.96 (1.09)	347 (69)
	Q4	246	1.20 (1.04)	1.98 (1.08)	352 (74)
	P-value	-	0.7	0.9	0.5

*C. pneumoniae*	Negative	288	1.14 (1.04)	1.90 (1.08)	349 (77)
	Positive	697	1.16 (1.02)	2.02 (1.05)	347 (72)
	P-value	-	0.6	0.5	0.7
	T1-T2	534	1.12 (1.03)	1.99 (1.05)	346 (72)
	T3-T4	175	1.32 (1.06)	2.11 (1.11)	353 (72)
	P-value	-	0.005	0.6	0.2

Presented as geometric mean (SE) for IL-6 and CRP; mean (SD) for fibrinogen

Q1-Q4 represents lowest through highest quartiles for all pathogens except *C. pneumoniae*

T1-T4 represents titer groups for *C. pneumoniae*

P-values by one-way ANOVA

*Cut-offs for seropositive status: cytomegalovirus≥ 8.0 Elisa Units (EU)/mL; herpes simplex virus-1 ≥ 16.0 EU/mL; hepatitis A virus, rate≤ 200; *H. pylori*≥ 0.90 EU/mL; *C. pneumoniae*≥ 1.0 Microimmunofluorescence (MIF) units

^†^High antibody response defined as top quartile of antibody response to pathogen (lowest quartile for hepatitis A virus) and titer groups 3-4 for *C. pneumoniae*. Low-intensity defined as bottom three quartiles to pathogens and titer groups 0-2 for *C. pneumoniae*.

**Figure 1:**
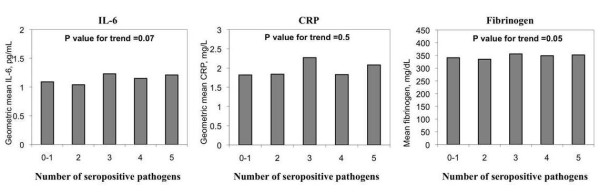
**Levels of interleukin-6 (IL-6), C-reactive protein (CRP) and fibrinogen by seropositive pathogen burden in a sub-sample of the Multi-Ethnic Study of Atherosclerosis (2000-2002)**. P values for trend by linear regression.

**Figure 2:**
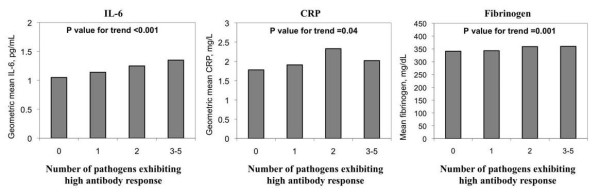
**Levels of interleukin-6 (IL-6), C-reactive protein (CRP) and fibrinogen by high antibody response to multiple pathogens in a sub-sample of the Multi-Ethnic Study of Atherosclerosis (2000-2002)**. P values for trend by linear regression.

Figures 1 and 2 show unadjusted inflammatory levels by seropositive pathogen burden and high antibody response to multiple pathogens, respectively. Seropositive pathogen burden was associated with higher circulating levels of IL-6 and fibrinogen but the associations were of borderline significance (p = 0.07 and 0.05 for trend, respectively). There was no association with CRP. The examination of associations of high antibody levels with inflammatory markers revealed stronger patterns. Levels of IL-6 (p < 0.001), CRP (p = 0.04) and fibrinogen (p = 0.001) were all significantly and positively associated with high antibody response to multiple pathogens.

Table 3 shows percent differences in inflammatory markers associated with both methods of assessing pathogen burden; seropositive pathogen burden and high antibody response to multiple pathogens, respectively, in four sequentially adjusted models. Positive associations between seropositive pathogen burden and IL-6 and fibrinogen were modest in model 1 and not statistically significant in adjusted models. In contrast, high antibody response to multiple pathogens was positively and significantly associated with IL-6, CRP and fibrinogen in model 1, with a 2 to 9% increase in inflammatory marker level per pathogen exhibiting a high antibody response. Estimates weakened with further adjustment, but associations with IL-6 remained statistically significant after adjustment for age, sex, race/ethnicity, and education (approximately 4% increase per pathogen exhibiting a high antibody response; model 3). Associations were reduced and no longer statistically significant when further adjusted for BMI, behavioral, and health variables (model 4).

**Table 3:** Percent difference in inflammatory markers associated with increasing seropositive pathogen burden* and increasing number of pathogens exhibiting high antibody response^† ^in a sub-sample of the Multi-Ethnic Study of Atherosclerosis (2000-2002).

Method	Inflammatory marker	Model 1	Model 2	Model 3	Model 4
Seropositive pathogen burden*	IL-6	2.2 (-0.9,5.3)	0.7 (-2.9,4.4)	-1.4 (-5.0,2.4)	-2.6 (-6.0,0.9)
					
	CRP	1.4 (-4.1,7.2)	0.5 (-5.9,7.3)	-2.0 (-8.4,4.9)	-4.2 (-10.1,2.0)
					
	Fibrinogen	0.7 (-0.3,1.6)	-1.2 (-2.3,0.0)	-1.6 (-2.7,-0.5)	-1.7 (-2.8,-0.6)

High antibody response to multiple pathogens^†^	IL-6	9.3 (4.9,13.8)	5.8 (1.5,10.3)	4.4 (0.0,8.9)	1.7 (-2.4,6.0)
					
	CRP	8.0 (0.2,16.4)	4.3 (-3.3,12.5)	2.2 (-5.3,10.4)	-0.2 (-7.3,7.4)
					
	Fibrinogen	2.0 (0.7,3.3)	0.7 (-0.6,2.0)	0.4 (-0.9,1.8)	0.2 (-1.1,1.5)

*Seropositive pathogen burden: seropositivity to 0-5 pathogens analyzed as a continuous variable.

^†^High antibody response to multiple pathogens: high antibody response to 0-5 pathogens analyzed as a continuous variable.

Coefficient (95% CI) shows the percent difference in inflammatory marker level per additional seropositive pathogen or per additional pathogen exhibiting a high antibody response.

Model 1 adjusted for age and sex

Model 2 adjusted for age, sex and race/ethnicity

Model 3 adjusted for age, sex, race/ethnicity and education

Model 4 adjusted for all of the above plus body mass index, alcohol intake, smoking (pack years), diabetes, medications, self-rated health.

Interactions of sex and race with pathogen burden (according to seropositivity or high antibody response) revealed no consistent patterns.

## Discussion

This is among the first studies to evaluate the relationship between pathogen burden based on conventional cut-offs for seropositivity and high antibody response as potential correlates of multiple markers of inflammation. We found that high antibody response was a more consistent marker of inflammatory outcomes than seropositivity alone. Previous work has generally not differentiated between seropositivity and antibody response [29], but our results suggest that this may be an important distinction. Earlier studies showed similar associations with inflammation in many of the pathogens examined here, suggesting that our results are compatible with population based estimates [8,26,30-32]. Also in line with previous findings, sociodemographic and health indices were significantly associated with number of seropositive pathogens [28,33-35]. There are less data, however, showing the relationships between sociodemographic characteristics and antibody response in population based studies in the US.

Testing of persistent pathogens often relies on enzyme linked immunosorbent assays (ELISA). These assays provide a qualitative assessment of the amount of antibody in a serum sample. Clinical studies are generally concerned with whether or not individuals tests positive for IgM or IgG to the pathogen of interest and rarely provide data on the qualitative antibody response identified in the ELISA test. Our findings regarding associations of antibody response with inflammation suggests that the qualitative data obtained from an ELISA is an important variable given that this measure predicts inflammation levels. Our findings suggest that antibody levels among the infected, rather than seropositivity is a better marker of inflammatory levels.

Pathogen burden (number of seropositive pathogens) was more strongly associated with inflammation than was any one individual pathogen and seropositivity to pathogens was highly correlated. Pathogen burden has been implicated in cardiovascular disease in several different study populations [33,36]. It has been suggested that pathogen burden may not only be a predictor of coronary complications, as previously described, but that it may also be associated directly with the development of atherosclerotic plaques [24]. Zhu and colleagues have suggested that inflammation is one pathway by which these associations are linked [8]. Prasad *et al*. found that pathogen burden and CRP levels interacted significantly such that carriers of 4-5 pathogens and elevated CRP levels (> 0.5 mg/dL) had higher odds of coronary artery disease compared to those with fewer pathogens [4], supporting the notion that pathogen burden along with inflammation has a stronger impact than any one pathogen alone or inflammation alone. A recent study showed that antibody levels to one of the pathogens we examined here, CMV, was associated with an increased rate of cardiovascular mortality among aging individuals [37]. No previous studies have examined the impact of high antibody response to multiple pathogens on inflammatory outcomes.

We found that associations of antibody levels with IL-6 were more consistent than associations with the other inflammatory markers. In addition, associations with IL-6 persisted after adjustment for age, race and socioeconomic factors but were reduced and no longer statistically significant after adjustment for potential mediators of inflammation such as smoking and obesity. Limited sample size, as well as strong associations between antibody levels and variables such as BMI and smoking may have hampered our ability to detect associations after multiple adjustments. Larger studies are needed to determine whether associations are independent of other risk factors.

There are several possible pathways by which high antibody response to multiple pathogens could impact inflammation more strongly than seropositivity alone. Infection with a particular pathogen may not strongly influence a disease process whereas a strong immune response to that pathogen may. Rheumatic heart disease is an example of this phenomenon whereby the immune response to the pathogen (and ensuing inflammation) is the relevant disease process, rather than the infection itself [38]. In the case of infections occurring early in life and persisting over time (likes several of the pathogens we studied); a higher initial infectious dose may lead to a more vigorous antibody response at a young age which is maintained over the life course for persistent infections. Since persistent infections are generally acquired early in life [39], the long term effects of high antibody response may lead to chronically higher levels of inflammation. However, it is not clear why individuals exhibit differentials in antibody response to pathogens. Recent work has indicated that lower socioeconomic position and exposure to chronic stressors may lead to a higher antibody response to CMV, HSV, and *H. pylori *[28,40]. Indeed, there are several experimental studies that have demonstrated significant relationships between exposure to psychosocial stressors and a high antibody response to persistent herpes virus (see Herbert and Cohen 1993 for review). Thus, a number of host characteristics may influence antibody response to an infection. Additional studies are needed to better understand the mechanisms by which antibody response may influence systemic inflammation over the life course.

Some methodological considerations of this study warrant mention. Relatively small sample sizes may have affected our ability to detect associations with all markers. The inflammatory outcomes we tested could have been influenced by a number of factors that were not measured or measured imprecisely, even though care was taken to account for several potentially inflammatory conditions and medications that may impact inflammation. Our models also did not account for recent or current acute inflammatory states, such as those associated with the common cold, which may have impacted our findings. Antibody and biomarker levels were only measured once and intra-individual variation cannot be accounted for. However, assay variability would be expected to bias findings towards the null so the observed associations are potentially underestimations. Also, IgG antibody response provides data on past infection- we did not examine data on recent infection or DNA shedding of the viruses and cannot assess whether individuals were experiencing reactivation of the infection. The cross-sectional nature of this analysis precludes implications of causality, and specifically, we cannot rule out the possibility that higher inflammatory status may have led to higher levels of antibodies via activation of the immune response. Four racial/ethnic groups were represented in our data, adding an important strength to our study; however, findings may not be representative of other ethnic or age groups. Finally, it is important to note that viral and bacterial infectious agents may act through different mechanistic pathways to impact inflammation, but assessment of alternative biological pathways, such as infection residing within the cardiovasculature, was beyond the scope of this cross-sectional observational study.

## Conclusions

In summary, high antibody response to multiple pathogens emerged as a better marker of inflammation compared to seropositivity status alone. Our findings suggest that future studies should consider including antibody response data in addition to seropositivity classification of pathogens. Technological advances for refining measurement of antibody response for persistent pathogens is an important endeavor and may reduce measurement error in this potentially important biomarker. Opportunities for further research also include examination of the mechanistic pathways by which antibody response to pathogens contributes to inflammation as well as to cardiovascular disease and its correlates.

## Competing interests

The authors declare that they have no competing interests.

## Authors' contributions

AN, AEA, and ADR drafted the manuscript and AN performed the statistical analysis. NSJ and MYT conducted the biochemical assays. ADR and MS provided important analytical feedback. All authors critically reviewed and commented on previous versions. All authors read and approved the final manuscript.

## Pre-publication history

The pre-publication history for this paper can be accessed here:

http://www.biomedcentral.com/1471-2458/10/706/prepub
